# *Helichrysum italicum*: From Extraction, Distillation, and Encapsulation Techniques to Beneficial Health Effects

**DOI:** 10.3390/foods12040802

**Published:** 2023-02-13

**Authors:** Veronika Furlan, Urban Bren

**Affiliations:** 1 Faculty of Chemistry and Chemical Engineering, University of Maribor, Smetanova 17, SI-2000 Maribor, Slovenia; 2 Faculty of Mathematics, Natural Sciences and Information Technologies, University of Primorska, Glagoljaška 8, SI-6000 Koper, Slovenia; 3 Institute of Environmental Protection and Sensors, Beloruska Ulica 7, SI-2000 Maribor, Slovenia

**Keywords:** *Helichrysum italicum*, polyphenolic compounds, biological effects, extraction methods, distillation methods, encapsulation methods

## Abstract

*Helichrysum italicum* (family Asteraceae), due to its various beneficial health effects, represents an important plant in the traditional medicine of Mediterranean countries. Currently, there is a renewed interest in this medicinal plant, especially in investigations involving the isolation and identification of its bioactive compounds from extracts and essential oils, as well as in experimental validation of their pharmacological activities. In this paper, we review the current knowledge on the beneficial health effects of *Helichrysum italicum* extracts, essential oils, and their major bioactive polyphenolic compounds, ranging from antioxidative, anti-inflammatory, and anticarcinogenic activities to their antiviral, antimicrobial, insecticidal, and antiparasitic effects. This review also provides an overview of the most promising extraction and distillation techniques for obtaining high-quality extracts and essential oils from *Helichrysum italicum,* as well as methods for determining their antioxidative, antimicrobial, anti-inflammatory, and anticarcinogenic activities. Finally, new ideas for in silico studies of molecular mechanisms of bioactive polyphenols from *Helichrysum italicum*, together with novel suggestions for their improved bioavailability through diverse encapsulation techniques, are introduced.

## 1. Introduction

The interest in natural phytochemicals concerning their therapeutic and beneficial health properties has gradually increased in recent years. Mediterranean plants are a rich source of bioactive compounds important to human health [1,2]. The genus *Helichrysum (Miller)* belongs to the Asteraceae family and includes more than a thousand taxa that have a high occurrence in the Mediterranean areas of Europe [3,4,5]. *Helichrysum (Miller)* grows at a wide range of altitudes from the sea level up to 1700 m, preferably on sandy or loamy soils [6]. The name of the genus is derived from the Greek words “helios” (sun) and “chryos” (gold) and is directly related to the typical bright yellow-colored inflorescences [6].

*Helichrysum italicum*, belonging to the *Helichrysum (Miller)* genus, is an evergreen plant native to the Mediterranean area. *Helichrysum italicum*, due to its various beneficial biological effects, represents an important everlasting plant in the traditional medicine of Mediterranean countries [5]. The interest in *Helichrysum italicum,* also known as immortelle or everlasting, has been motivated by its traditional therapeutic applications in inflammatory and allergy conditions, such as asthma and skin inflammatory conditions [7]. The use of *Helichrysum italicum* essential oils has also been reported in aromatherapy applications, wound healing, and skin conditions such as hematoma and sunburn [8]. Voinchet et al. [9] showed that the application (for 2–3 months) of *Helichrysum italicum* subsp. *serotinum* essential oil diluted to 10% in *Rosa rubiginosa* vegan oil reduced local inflammation, edema, bruises, and hematomas in the post-operative scars. In addition, its therapeutic use, related to antioxidant and antimicrobial properties [10,11,12,13] has long been recognized. In the agri-food sector, *Helichrysum italicum* flowers can be used for seasoning and flavoring food, such as bakery products and soft drinks, and as natural food additives or preservatives due to their antibacterial (against *Micrococcus luteus*, *Bacillus cereus*, and *Pseudomonas aeruginosa*) [14], antifungal (against *Aspergillus niger* and *Alternaria alternata*) [14] and insecticidal properties (against mosquito *Aedes albopictus* (Diptera: Culicidae)) [15]. In a very recent study, the consumption of *Helichrysum italicum* infusion was reported to significantly reduce serum levels of proinflammatory interleukine 1β (IL-1β) alongside Proteobacteria reduction. According to the authors, *Helichrysum italicum* infusion possesses prebiotic activities and can improve gut microbiota [16].

Currently, there is renewed interest in this medicinal plant in investigations involving isolation and identification of bioactive compounds, and in the experimental validation of their pharmacological activities. In this paper, we review the current knowledge on the beneficial health effects of *Helichrysum italicum* extracts, essential oils, and their major bioactive compounds. Moreover, this review summarizes the knowledge on the most promising extraction and distillation techniques for obtaining high-quality extracts and essential oils from *Helichrysum italicum,* as well as methods for determining their antioxidative, antimicrobial, anti-inflammatory, and anticarcinogenic activities. In addition, new ideas for in silico studies of molecular mechanisms of bioactive polyphenols from *Helichrysum italicum*, together with diverse encapsulation techniques for their improved bioavailability, are introduced.

### Identification and Taxonomic Classification of Helichrysum italicum

**Figure 1:**
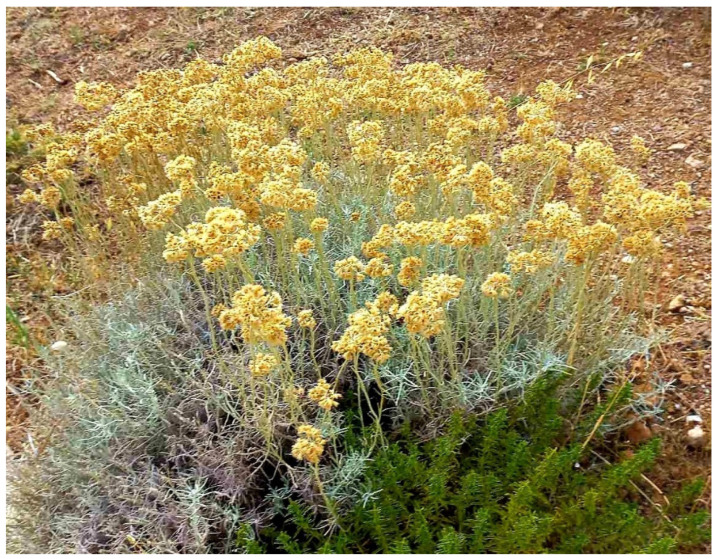
*Helichrysum italicum* in full blossom (photo taken by Dr. Veronika Furlan).

*Helichrysum italicum,* synonymously immortelle, or everlasting, is a 30–70 cm high aromatic shrub with small yellow flowers that exhibit a strong and persistent smell similar to curry [10]. It grows widely in natural, dry, and sandy-rocky areas of Mediterranean regions, and is adapted to survive in environments that lack water [5,17,18]. More recently the native Mediterranean plant of immortelle has been found to possess numerous phytochemicals with various biological effects, namely anti-inflammatory [19], antimicrobial [20], antioxidant [12,21], antiviral [22], anti-HIV [10], antilarvicidal, and repellent activities [23]. Known therapeutic applications include the healing of wounds, treating gall and bladder disorders, and analgesic uses [17]. Nowadays, valuable beneficial health effects of plant extracts and essential oils are becoming of high economic relevance, and *Helichrysum italicum* cultivation is widely spread in Corsica, Italy, Hungary, Bosnia and Herzegovina, and Croatia. *Helichrysum italicum* in full blossom is shown in Figure 1.

Viegas et al. [5] presented a very complex taxonomic classification of *Helichrysum italicum* into six subspecies (subsp.), which are distributed in biodiversity hotspots of Mediterranean countries:

(1) subsp. *italicum* (Corsica, Italy, Cyprus, isolated localities in Morocco)
(2) subsp. *microphyllum* (Willd.) Nyman (Balearic Islands, Sardinia, Corsica, Crete, and Cyprus)
(3) subsp. *picardii* (France, Italy, Portugal, and Spain)
(4) subsp. *pseudolitoreum* (Argentario, Gargano, and Mount Conero)
(5) subsp. *serotinum* (Iberian Peninsula)
(6) subsp. *siculum* (Sicily) [5].

A recent study by Herrando-Moraira et al. [24] proposed a revised taxonomic classification for the *Helichrysum italicum* into four subspecies:

(1) subsp. *italicum* (Italy, Croatia, eastern Mediterranean coast of France and Corsica, Bosnia and Herzegovina, Greece -Aegean islands and Cyprus),
(2) subsp. *microphyllum* (Crete),
(3) subsp. *siculum* (Sicily), and
(4) subsp. *tyrrhenicum* (Corsica, Sardinia, Majorca, and Dragonera Islet).

*Helichrysum italicum* extracts and essential oils possess a wide variety of chemical classes, among which dominate flavonoids, α-pyrones, phenolic acids, acetophenones, tremetones, monoterpenes, sesquiterpenes, and triterpenes [25]. It is important to underline that a comparison of the chemical composition of essential oils and extracts from different regions of the Mediterranean basin demonstrated different compositions of bioactive compounds among samples [17]. The main factors influencing the composition of plant extracts and essential oils are the environmental characteristics of growing sites (ecology, climate, and geographical location), the developmental stage of the plant, texture, and acidity of soils, and the plant’s genotype or subspecies [23,26,27].

The authentication of the collected *Helichrysum italicum* plant is the crucial step before the isolation and subsequent analysis of obtained extracts and essential oils. Recently, the DNA barcoding methodology is being implemented to characterize, differentiate, and identify the plants from which essential oils and extracts were obtained. It is based on sequencing specific gene regions (barcodes), that exhibit high interspecies, and low intraspecies, variability. For the *Helichrysum* genus, the recommended markers are two plastidial genes, namely matK, and rbcL [28]. Additional gene markers, namely internal transcribed spacer 1 and 2 (ITS1/2) and the plastid trnH-psbA intergenic spacer have been reported to improve further discrimination. De Mattia et al. [29] reported that matK and rbcL markers represent the most suitable combination for the Asteraceae family, whereas matK represents the most successful single marker for this family. It was concluded that these two markers can successfully characterize plants from the family Asteraceae at the genus level. At the time of writing this review, the largest barcode reference BOLD [30], contained *Helichrysum italicum* barcodes for matK, rbcL, ITS1, and ITS2 markers; however, the trnH-psbA barcode was not available.

Systematic cultivation of immortelle demands the identification of genetic material at the subspecies level for further target-oriented breeding, stable quality, and yield of valuable secondary metabolites in *Helichrysum italicum* extracts, and essential oils at the industrial level. Baruca Arbeiter et al. [31] recently developed the first set of microsatellite polymorphisms, which represent valuable DNA markers and a promising opportunity for the selection of the most promising genotypes of *Helichrysum italicum* subspecies for further breeding programs, propagation, and their implementation in agricultural production. Research of genotypes of numerous populations of Mediterranean *Helichrysum italicum*, coupled with knowledge of their chemical composition (chemotype), could, therefore, provide valuable information about prospective genotypes/chemotypes for the pharmaceutical, cosmetic, and food industries.

## 2. Extraction, Distillation, and Analytical Methods for Obtaining Extracts, Essential Oils as Well as Individual Bioactive Compounds from *Helichrysum italicum*

**Figure 2:**
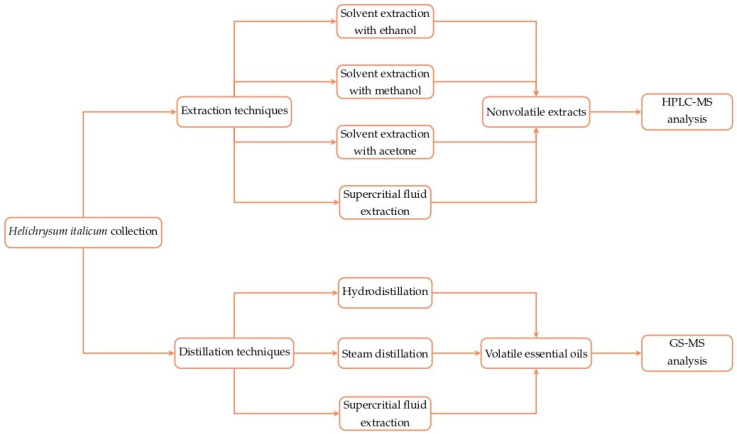
Pipeline process from *Helichrysum italicum* collection to the identification of bioactive compounds.

The choice of isolation and extraction method significantly affects the composition of obtained *Helichrysum italicum* essential oils and extracts. The pipeline process from the *Helichrysum italicum* collection to the identification of bioactive compounds is presented in Figure 2.

The applied organic solvent extraction and distillation techniques for obtaining *Helichrysum italicum* extracts and essential oils enriched with major bioactive compounds, together with identification methods, are presented in Table 1.

*Helichrysum italicum* essential oils (EOs) are produced from the glandular hairs present on its leaves and flower heads by hydrodistillation and steam distillation of early flowering tops (cut by hand from mid-June to mid-July) [23]. Although hydrodistillation is the most frequently used method for obtaining EOs from *Helichrysum italicum*, it is important to know that the use of high temperatures can affect the quality of obtained essential oils [32]. The low content of essential oil in the plant (0.2–0.3%) contributes to its extremely high price (30–120 €/5 mL), as a ton of flowering tops produces only about 900 g to 1.5 kg of essential oil. The chemical composition of commercialized essential oils is usually determined by gas chromatography coupled with mass spectrometry (GC/MS) and flame ionization detector (GC/FID) analysis. As can be observed from Table 1, the reported chemical profiles of *Helichrysum italicum* essential oil obtained by hydrodistillation and steam distillation indicate the dominance of monoterpenes α-pinene, limonene, nerol, neryl acetate, and neryl propanoate, as well as sesquiterpenes α-selinene, β-selinene, and γ-curcumene.

On the other hand, organic solvents are most frequently used for obtaining *Helichrysum italicum* extracts rich in polyphenols (flavonoids, pyrones, acetophenones, tremetones, phenolic acids, and their esters), followed by lipids (santiols and sitosterols) [32]. The most commonly applied organic solvents are ethanol and methanol, followed by acetone, while the chemical composition of the obtained extracts is usually determined by high-performance liquid chromatography (HPLC). The major bioactive compounds in *Helichrysum italicum* extracts obtained by organic solvents were found to be a prenylated phloroglucinyl α-pyrone arzanol, flavonoids gnaphaliin, tiliroside, naringenin, and pinocembrin, phenolic acids chlorogenic and caffeic acid, acetophenones, tremetones, and triterpene ursolic acid (Table 1).

**Table 1:** Isolation techniques for obtaining *Helichrysum italicum* extracts and essential oils enriched with bioactive compounds.

Compound Class	Compounds	Isolation Techniques	Identification Methods	References
**Essential oils**				
**Monoterpenes**	α-pinene, limonene, nerol, neryl acetate, and neryl propanoate	hydrodistillation with Clevenger-type apparatus	GC-FID, GC-MS	[15,26,27,33,34,35,36,37,38,39,40,41,42,43]
		steam distillation with spring-type apparatus	GC-FID, GC-MS	[4,6]
**Sesquiterpenes**	α–selinene, β-selinene, γ-curcumene, and eudesm-5-en-11-ol	hydrodistillation with Clevenger-type apparatus	GC-FID, GC-MS	[40,44,45,46,47,48]
		steam distillation with spring-type apparatus	GC-FID, GC-MS	[4]
**Extracts**				
**Polyphenolic acids**	chlorogenic acid, caffeic acid	accelerated solvent extraction using methanol-water (3:1)	HPLC-MS/MS	[49]
		solvent extraction using methanol	HPLC, HRESIMS/MS, 1H NMR, 13C NMR, DQF-COSY	[50]
		solvent extraction using methanol	HPLC-DAD	[39]
		solvent extraction using ethanol	UV-VIS, IR, MS	[51]
			HPLC, 1H NMR	[52]
**Flavonoids**	gnaphalin, tiliroside, pinocembrin	solvent extraction using methanol	Gravity column chromatography on silica gel, UV, IR, 1H NMR, 13C NMR, HRESIMS	[21,53,54]
	naringenin, kaempferol, quercetin	accelerated solvent extraction using methanol-water (3:1)	HPLC-MS/MS	[49]
		solvent extraction using methanol	HPLC, HRESIMS, 1H NMR, 13C NMR, DQF-COSY	[50]
	gnaphalin, naringenin, apigenin, luteolin, kaempferol, quercetin	solvent extraction using ethanol (70%)	UV-VIS, EI-MS, FD-MS	[3]
			HPLC, UV-VIS	[55]
			MECC-DAD, HPLC-DAD, UV-VIS	[56]
**Pyrones**	arzanol	solvent extraction using acetone	Gravity column chromatography on silica gel, HPLC, HRESIMS, 1H NMR, 13C NMR, IR, UV	[10,12,13]
**Acetophenones**	4-hydroxy-3-(2-hydroxy-3-isopentenyl)acetophenone 4-hydroxy-3-(3-methyl-2-butenyl) acetophenone	solvent extraction with methanol	gravity column chromatography on silica gel, UV, IR, 1H NMR, 13C NMR, HRESIMS	[53]
			TLC, HPLC-DAD	[21]
**Tremetones**	12-acetoxytremetone	solvent extraction with ethanol	Gravity column chromatography on silica gel, HPLC, ESI-MS, UV, IR, 1H NMR, 13C NMR, DQF-COSY	[57,58]
		solvent extraction with acetone	Gravity column chromatography on silica gel, HRESIMS, 1H NMR, 13C NMR, IR, UV	[10,12]
	12-hydroxytremetone	solvent extraction with methanol	Gravity column chromatography on silica gel, UV, IR, 1H NMR, 13C NMR, HRESIMS	[53]
**Triterpenes**	ursolic acid	solvent extraction with methanol	Gravity column chromatography on silica gel, UV, IR, 1H NMR, 13C NMR, HRESIMS	[53]
			TLC, HPLC-DAD	[21]
		solvent extraction with acetone	Gravity column chromatography on silica gel, HPLC, HRESIMS, 1H NMR, 13C NMR	[13]

Although the European Union (EU) lists ethanol, acetone and methanol as acceptable solvents for the isolation of polyphenolic compounds from plant materials [59], the main disadvantage of using organic solvent extraction methods is the need for further separation of extracted compounds with fractionation agents (namely petroleum ether, dichloromethane, ethyl acetate, diethyl ether, n-hexane, methanol) and the remaining organic solvent traces in the extracts. Therefore, recent studies applied the supercritical CO_2_ extraction (SFE) technique to obtain non-toxic, solvent-free extracts at low temperatures with high extraction yields and higher selectivity of the extracted compounds from *Helichrysum Italicum* [60,61]. An overview of extraction conditions, extraction yields, and major bioactive compounds from supercritical CO_2_ extracts, together with applied identification methods, is presented in Table 2.

As can be observed from Table 2, the most common monoterpenes present in volatile fractions of supercritical *Helichrysum Italicum* extracts are similar to those obtained by hydro and steam distillation: α-pinene, nerol, neryl acetate, and neryl propanoate. Moreover, it was reported that *Helichrysum Italicum* supercritical CO_2_ extract is dominated by sesquiterpenes, namely α-selinene, β-selinene and γ-curcumene, and also contained significant content of waxes [60,62].

An important advantage of supercritical CO_2_ extraction is its ability to isolate both volatile essential oils and non-volatile phenolic compounds from the plant material. Jokic et al. [67] performed supercritical fluid extraction of scopoletin from *Helichrysum. italicum* and reported that the highest yield of scopoletin (6.31%) was obtained under the extraction conditions of 200 bar and 40 °C. Scopoletin was determined using reversed-phase HPLC with UV detection.

In a very recent article, Maksimovic et al. [66] analyzed the chemical profiles of *Helichrysum italicum* extracts obtained by SFE, with and without cosolvent ethanol, using GC-FID, GC-MS, HPLC, and UHPLC-MS techniques. The SFE of *Helichrysum italicum* was performed under 350 bar and 40 °C, producing extracts with a high content of bioactive sesquiterpenes (α-selinene, β-selinene, and γ-curcumene derivatives) and phenolic flavonoids (pyrogallol and chlorogenic acid derivatives). The addition of ethanol as a cosolvent influenced both the solubility power and selectivity of supercritical CO_2_, which resulted in almost doubled extraction yield. Moreover, the authors were the first to identify the presence of arzanol in supercritical CO_2_ extracts of *Helichrysum italicum*, which represents a valuable basis for further studies of its molecular mechanisms and pharmacological activities.

However, except for these studies [66,67], there are no reports on HPLC analysis of *Helichrysum italicum* supercritical extracts obtained at higher pressures or with the use of polar cosolvents, such as methanol, ethanol, and acetone, that could enhance the extraction of polar phenolic compounds. Moreover, there is an evident lack of studies addressing the biological activities of supercritical extracts of various *Helichrysum italicum* subspecies, which are limited to antimicrobial and antioxidant activities tested in vitro. Therefore, the isolation of polyphenolic compounds by supercritical CO_2_ with the addition of a cosolvent, as well as their analysis with appropriate identification methods, such as HPLC, should be further explored.

## 3. Methods and Techniques for Determining Biological Effects of Extracts, Essential Oils as Well as Individual Bioactive Compounds from *Helichrysum italicum*

### 3.1. Methods and Techniques for Determining Antioxidative Effects

Spectrophotometric methods the 2,2-diphenyl-1-picrylhydrazil (DPPH), and the 2,2’-azino-bis(3-ethylbenzothiazoline-6-sulfonic acid) (ABTS) assays are commonly used for the in vitro determination of the antioxidative activity of *Helichrysum italicum* extracts and essential oils. The obtained efficient concentration (EC_50_) value represents a concentration of the tested compound that causes a 50% reduction in spectrophotometric absorbance. The reduction of the color of the measured reagent is proportional to the concentration and antioxidative activity of the tested compound [68,69]. DPPH is mainly used to measure the overall free radical scavenging activity of *Helichrysum italicum* extracts, essential oils, or individual compounds, as it is performed at ambient temperatures, thereby the degradation of several thermolabile polyphenolic compounds, namely arzanol, tiliroside, and gnaphaliin, can be avoided [12,21,70].

*Helichrysum italicum* flavonoids and terpenes are also effective in inhibiting lipid peroxidation, which indicates cell membrane injury. The inhibition of Fe^2+^/ascorbate system-induced lipid peroxidation by the tested antioxidant is determined by measuring the formation of malondialdehyde (MDA), which is a low-molecular-weight end product of the decomposition of various primary and secondary lipid peroxidation metabolites [54,71,72]. The results are expressed by the inhibitory concentration (IC_50_), which indicates the required concentration of the tested compound to inhibit lipid peroxidation by 50% [73].

### 3.2. Methods and Techniques for Determining Antimicrobial Effects

Dilution or diffusion methods are most commonly used to measure in vitro antimicrobial activities of bioactive compounds from *Helichrysum italicum* [74]. The antimicrobial activity of the tested compound is evaluated by measuring the minimum inhibitory concentration (MIC) and minimum bactericidal concentration (MBC). MIC represents the lowest concentration of the tested compound that inhibits the visible growth of a microorganism after incubation, while MBC represents the lowest concentration of the tested compound that results in the death of the microorganism [75,76].

The quantitative determination of antimicrobial activity by MIC and MBC values can be achieved by diffusion methods, as well as by both agar dilution and broth dilution methods. When diffusion methods are used to measure the antimicrobial activity of *Helichrysum italicum* essential oils, the absence of visible inhibition zones may arise because of the troublesome diffusion of nonpolar monoterpenes through the culture medium. However, this does not necessarily mean that the tested essential oil is inactive against the microorganism. Analogously, when using agar dilution and broth dilution methods, water solubility problems of hydrophobic terpenes (e.g., α-pinene) from essential oils can arise. To overcome the solubility issues, DMSO or detergent Tween 80 is used to initially solubilize the tested hydrophobic compounds [14,34,77,78].

### 3.3. Methods and Techniques for Determining Anticarcinogenic Effects

In vitro assays, namely the 3-(4,5-dimethylthiazol-2-yl)-2,5-diphenyltetrazolium bromide (MTT) assay and the comet assay, are commonly used for quantitative evaluation of anticarcinogenic activities of bioactive compounds from *Helichrysum italicum.*

The MTT assay is used to measure the cytotoxic or cytostatic activities of bioactive compounds from *Helichrysum italicum* [79,80]. In viable tumor cells, MTT is reduced into the colored substance formazan, which can be measured spectrophotometrically. The anticarcinogenic potential of the tested compound is expressed by the concentration causing a 50% growth inhibition or death of tumor cells (IC_50_). A lower IC_50_ value indicates that the tested compound is better at inhibiting or killing tumor cells.

The comet assay, also termed single-cell gel electrophoresis, is a method for evaluating DNA damage in eukaryotic cells [81,82,83]. The assay can be performed in in vitro, ex vivo, and in vivo systems by concurrently exposing eukaryotic cells to a known genotoxic agent (e.g., hydrogen peroxide) and the investigated compound with antigenotoxic potential (e.g., arzanol from *Helichrysum italicum*). Electrophoresis at high pH values results in structures resembling comets, where the lower intensity ratio between the comet tail and head indicates that the tested antigenotoxic compound (e.g., arzanol) more effectively protects the cells from DNA damage induced by a genotoxic agent (e.g., hydrogen peroxide). The magnitude of DNA damage/protection of the tested tumor cells can be assessed by comparing the obtained intensity ratios with standards (e.g., X-ray irradiated cells).

### 3.4. Methods and Techniques for Determining Anti-Inflammatory Effects

The most common in vivo studies of *Helichrysum italicum* extracts and essential oils include acute inflammatory response models [84]. The most studied in vivo models include carrageenan-induced paw edema and croton oil-induced ear edema models, induced pain models in rodents, the formalin test in mice, and the carrageenan-induced pleurisy mouse model [19,21,53,85]. Commonly injected pro-inflammatory substances to induce an inflammatory response in rodents include carrageenan, croton oil, lipopolysaccharide, formalin, and rotenone. To evaluate anti-inflammatory activity, rodents are subsequently treated with *Helichrysum italicum* extracts, essential oils, or their biologically active compounds (oral administration or intraperitoneal injection). The statistical evaluation of the obtained results is based on comparison with the control group not receiving *Helichrysum italicum* extracts or essential oil (statistical significance level at least *p* < 0.05) [11,19,21,53,57,86,87,88].

## 4. Biological Effects of *Helichrysum italicum* Extracts

*Helichrysum italicum* extracts, mainly produced from inflorescences, have high potential in the pharmaceutical, cosmetic, and food industries, as they exert several beneficial health effects, namely antibacterial, antioxidative, anti-inflammatory, as well as anticarcinogenic effects [20,34,89,90]. Nostro et al. [20] suggested that terpenes and flavonoids from a diethyl ether extract of *Helichrysum italicum* are mainly responsible for its antibacterial activity, and highlighted their prominent role in interactions with the cytoplasmatic membrane of *Staphylococcus aureus*, leading to the structural destabilization of the membrane. The authors also showed that there was no difference in inhibition of methicillin-resistant *Staphylococcus aureus* and methicillin-sensitive *Staphylococcus aureus* strain growth. It was also demonstrated that the diethyl ether extract of *Helichrysum italicum* at sub-minimum inhibitory concentrations (sub-MIC) from 62.5 to 125 μg/mL reduced the activity of *Staphylococcus aureus* enzymes DNAse, lipase, thermonuclease, and coagulase, as well as preventing the production of enterotoxins B and C [91]. In their later study Nostro et al. [92] inferred that the reduction of growth and cariogenic effects of *Streptococcus* mutants may occur due to the flavonoids present in the *Helichrysum italicum* ethanolic extract (MIC values from 31.25 to 62.50 μg/mL). Moreover, the antibacterial activity of *Helichrysum italicum* etanol-water extract (rich in caffeic acid (156.1 mg/L)), was tested on Gram-negative bacteria *Compylobacter coli, Escherichia coli*, and *Salmonella infantis*, as well as on Gram-positive *Bacillus cereus, Listeria monocytogenes,* and *Staphylococcus aureus*. The results revealed stronger antibacterial activity against multidrug-resistant Gram-positive bacteria (MIC values from 0.02 to 0.06 mg/mL), which indicates that *Helichrysum italicum* extracts represent an effective therapeutic alternative to conventional antibiotics [93]. Moreover, Tundis et al. [17] investigated the antibacterial activity of methanolic extracts of *Helichrysum italicum* from Calabria and Sardinia, which were the most effective against the Gram-positive bacteria *Micrococcus luteus* (MIC value of 50 μg/mL). The authors also reported that the Sardinian extract (rich in α-terpinolene, trans-caryophyllene, and neryl acetate) was more active against phytopathogen fungus *Pythium ultimum* than the Calabrian extract (growth inhibition up to 72.6% and 56.8%, respectively) probably due to the difference in the chemical composition between Calabrian and Sardinian *Helichrysum italicum* extracts. In addition, a diethyl ether extract obtained from the flowering tops of *Helichrysum italicum* was studied for its anti-herpes simplex virus (HSV-1) activity. According to the authors, diethyl ether extract was effective against HSV in concentrations ranging from 100 to 400 μg/mL [22]. It was also suggested that flavonoids were mainly responsible for the anti-HSV activity [5,94].

In a recent study, Molnar et al. [95] investigated different medicinal plant extracts and analyzed their DPPH radical scavenging capacity and total phenolic content. A 96% ethanolic extract of *Helichrysum italicum* possessed a high total phenol content (132.1 mg gallic acid equivalents/g), which was highly correlated with DPPH scavenging activity (93.5%). Furthermore, Sala et al. [19] reported that a *Helichrysum italicum* methanolic extract and its butanol (BuOH), ethyl acetate (EtOAc), hexane, and dichloromethane (CH_2_Cl_2_) fractions can significantly reduce 12-O-tetradecanoylphorbol-13-acetate (TPA)-induced edema in mice ears, with the butanol fraction being the most effective (*p* < 0.01 vs. untreated control). However, ethyl phenylpropiolate-induced acute ear edemas were significantly reduced only by ethyl acetate and butanol fractions enriched with high-polarity flavonoids and acetophenones. A methanolic extract, hexane fraction (enriched with lipids and sitosterol), and dichloromethane fraction (enriched with ursolic acid, 4-hydroxy-3-(3-methyl-2-butenyl)acetophenone, and gnaphaliin) at a dose of 200 mg/kg reduced TPA-induced chronic inflammation in the mouse ear by 65, 44, and 48%, respectively. The methanol extract and the butanol fraction also exhibited the highest anti-inflammatory activity against phospholipase A2 (PLA2)-induced acute edema in the mouse paw. Moreover, methanolic extract and its fractions reduced sheep red blood cell-induced delayed-type hypersensitivity in mouse paw by 40% to 66%. Finally, all the fractions significantly reduced the serotonin-induced acute edemas in the mouse paw, with the ethyl acetate fraction being the most effective. According to the authors, the observed difference in anti-inflammatory activity can be attributed to compounds of different polarity present in the methanolic extract of *Helichrysum italicum* and its fractions. Furthermore, Goncalves et al. [96] reported that the methanol extract of *Helichrysum italicum*, rich with phenolic compounds (caffeoylquinic and dicaffeoylquinic acids as well as pinocembrin), exhibited high inhibitory activity against enzymes involved in Alzheimer’s disease progression, namely acetylcholinesterase (AChE) (78.29%), tyrosinase (74.13%) and α-glucosidase (96.65%). In addition, the study of Rigano et al. [57] showed that the 30 μg/mL ethanolic extract of the *Helichrysum italicum* flowers induced antispasmodic actions in the isolated mouse ileum, and significantly inhibited motility in the inflamed gut, which confirmed the traditional use of the *Helichrysum italicum* ssp. *italicum* herbal tea as a cure for inflammatory bowel disease without toxic side effects.

It must be noted that *Helichrysum italicum* extracts represent a mixture of various compounds that can exert synergistic pharmacological activities as well as reduce the unwanted side effects of individual compounds. This complexity also results in challenging chemical analyses and evaluation concerning which compound or compound combination is responsible for specific biological activities. For specific therapeutic applications, herbal extracts must be standardized based on an active component [97]. However, European Pharmacopoeia and WHO do not provide specific guidelines for obtaining standardized extracts of *Helichrysum italicum* [98,99]. In the following subsections, we will summarise the already known biological effects of major bioactive compounds present in *Helichrysum italicum* extracts.

### 4.1. Biological Effects of Major Bioactive Compounds from Helichrysum italicum Extracts

*Helichrysum italicum* extracts contain mainly non-volatile polyphenolic compounds that possess various beneficial biological effects, namely antioxidative, anti-inflammatory, antimicrobial, and anticarcinogenic effects, with cytoprotective activity towards normal cells and cytotoxic effects against cancer cells [100].

Polyphenols are a large group of at least 10.000 known compounds which contain one or more aromatic rings with at least one phenolic hydroxyl group. They are secondary plant metabolites that protect the plants against reactive oxygen and nitrogen species, UV light, pathogens, and parasites [97,101].

**Figure 3:**
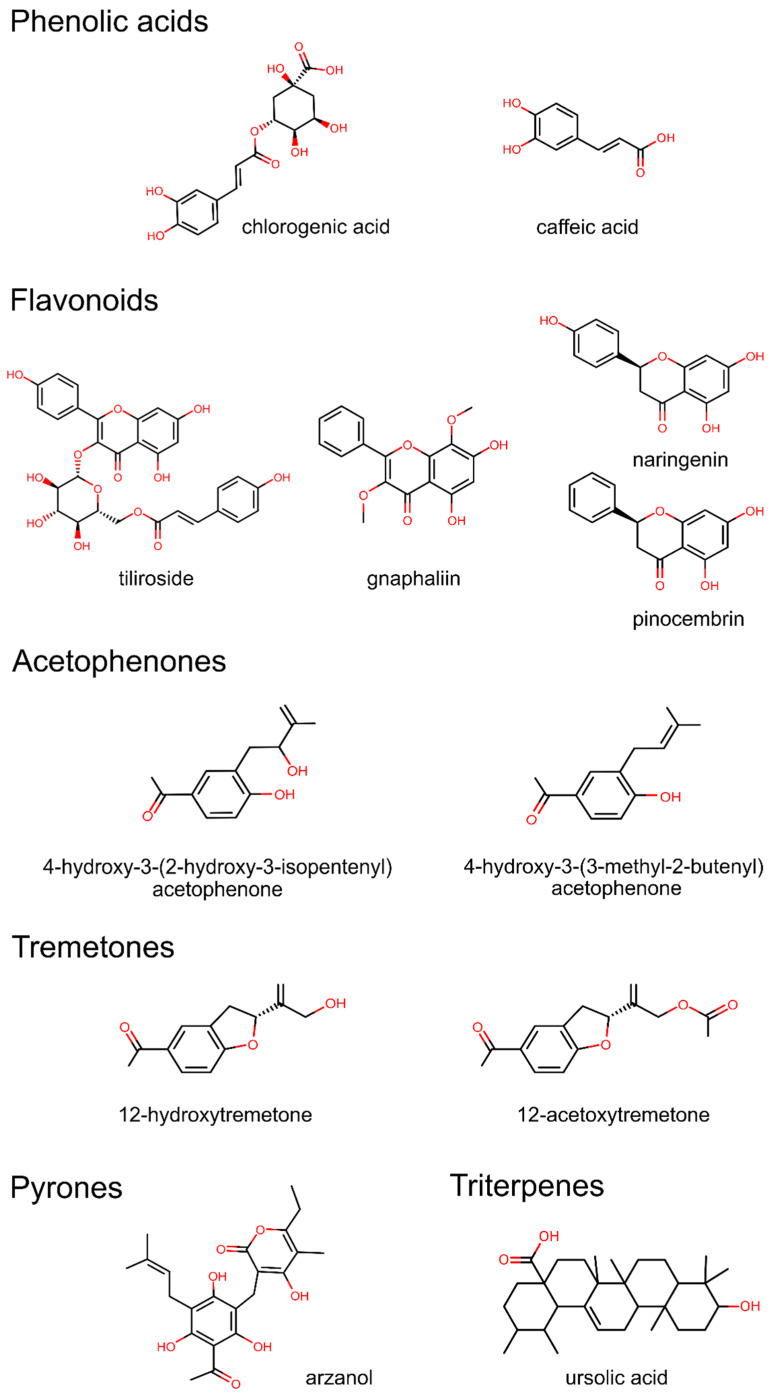
Major bioactive compounds from *Helichrysum italicum* extracts.

The quality of *Helichrysum italicum* extracts is correlated mainly with the content of flavonoids (e.g., gnaphaliin and tiliroside), and a prenylated α-pyrone–phloroglucinol heterodimer arzanol, as well as with the content of polyphenolic acids (e.g., chlorogenic acid), acetophenones (e.g., 4-hydroxy-3-(3-methyl-2-butenyl)acetophenone), and triterpenes (e.g., ursolic acid). The chemical structures of the main bioactive compounds found in *Helichrysum italicum* extracts are depicted in Figure 3. In the following subsections, we summarize the most pronounced biological effects of major bioactive compounds from *Helichrysum italicum* extracts.

#### 4.1.1. Phenolic Acids

Phenolic acids, containing a phenolic ring and a carboxylic acid functional group, can be divided into two groups, namely hydroxycinnamic and hydroxybenzoic acids with their respective derivatives [102]. Chlorogenic acid, an ester of caffeic and quinic acid, is the most abundant hydroxycinnamic acid from *Helichrysum italicum* methanolic extracts (up to 0.77% of the extraction yield) [51,103]. In vitro and in vivo studies have reported several pharmacological effects of chlorogenic acid, namely antioxidant, anti-inflammatory, anticancer, antibacterial, and antiviral effects.

Vanucci-Bacqué et al. [104] demonstrated the antioxidant activity of chlorogenic acid (10 μM), which was assessed as superoxide anion radical scavenging activity (35.5%). DPPH free radical scavenging activity of chlorogenic acid was also reported (IC_50_ 20 μg/mL) [105]. Moreover, Luyen et al. [106] reported anti-inflammatory activity of chlorogenic acid (10 uM) in mouse macrophage RAW264.7 cells, which was assessed as inhibition of lipopolysaccharide (LPS)-stimulated tumor necrosis factor (TNF-α) production (24.73%). Chlorogenic acid (100 uM) also inhibited cyclooxygenase 2 (COX2) by 30% [107]. The inhibition of the proliferation of human glioma U251 cancer cells (56.63%) and rat glioma C6 cancer cells (77.37%) by 100 uM chlorogenic acid was also observed [108]. Furthermore, D’Abrosca et al. [109] reported that chlorogenic acid (128 μg/mL), isolated from the methanol extract of *Helichrysum italicum,* inhibited biofilm formation of *Pseudomonas aeruginosa* by 45%. Konstantinopoulou et al. [110] also demonstrated the antimicrobial activity of chlorogenic acid against *Helicobacter pylori* (MIC 6.25 μg/mL). The antifungal activity of chlorogenic acid against *Candida krusei* and *Candida albicans* (MIC > 64 μg/mL) was observed as well [111]. In addition, it was reported that chlorogenic acid (25 μM) inhibited human immunodeficiency virus type 1 integrase (HIV-1 IN) by 59.7% [112].

Caffeic acid is a very common hydroxycinnamic acid with many beneficial biological effects, which is present in *Helichrysum italicum* methanolic extracts up to 0.015% [51,103,113]. In the study of Georgiev et al. [114], caffeic acid (3.6 mM) demonstrated 88.04% DPPH radical scavenging activity. Similarly, Digiacomo et al. [115] reported 90.27% DPPH radical scavenging activity caffeic acid (30 uM). Bora-Tatar et al. [116] identified caffeic acid (500 μM) as a potent histone deacetylase (HDAC) inhibitor due to its 80% inhibition of HDAC in human immortal Hela cells. Yu et al. [86] also reported significant inhibition of potato 5-lipoxygenase (5-LOX) by caffeic acid (4 μg/mL), indicating its anti-inflammatory activity. The authors also reported significant anti-inflammatory activity of caffeic acid (30 mg/kg) against carrageenan-induced paw edema in a rat model. The anti-inflammatory activity of caffeic acid was also assessed as inhibition of LPS-induced TNF-α (IC_50_ > 50 μg/mL), IL-12 (IC_50_ > 50 μg/mL), and IL-6 (IC_50_ > 50 μg/mL) production in wild-type embryonic C57BL/6 mouse bone marrow dendritic cells [117]. Moreover, the MTT assay of Chen et al. [118] confirmed the cytoprotective activity of caffeic acid against H_2_O_2_-induced cytotoxicity in human endothelial Ea.hy926 cancer cells (EC_50_ 12.6 μM). Miamaye et al. [119] also demonstrated inhibition of human amyloid beta (A42) aggregation by caffeic acid (IC_50_ 32.8 μg/mL), which indicates it has potential in the treatment of Alzheimer’s disease. Furthermore, caffeic acid (50 μg/mL) demonstrated antibacterial activity against *Fusarium graminearum* (63%) [120] and *Staphylococcus epidermidis* (EC_50_ 2.78 μg/mL) [121]. The MTT assay of Fu et al. [122] also showed its antifungal activity against *Candida albicans* (MIC > 50 μg/mL) as well as antibacterial activity against *Pseudomonas fluorescens* (MIC > 50 μg/mL), *Staphylococcus aureus* (MIC > 50 μg/mL) and *Bacillus subtilis* (MIC > 50 μg/mL). In addition, it was observed that caffeic acid inhibits HIV1 integrase strand transfer activity (IC_50_ 24 μg/mL) and, therefore, possesses antiviral activity [123].

#### 4.1.2. Flavonoids

Flavonoids are the largest group of dietary polyphenols. They possess a 15-carbon structure consisting of two phenyl rings and a heterocycle. Due to their structural diversity, they are further divided into seven subclasses; namely flavanols (catechins), flavanones, flavones, flavonols, isoflavones, anthocyanins, and chalcones. According to several studies, polyphenols from the flavonoid class possess antioxidant, anti-inflammatory, antiproliferative, anticarcinogenic, and antimicrobial activities [124]. Flavonols gnaphaliin and tiliroside, as well as the flavanone naringenin, are the most common flavonoids, present in *Helichrysum italicum* methanolic extracts up to 0.03%, 0.0063%, and 0.023%, respectively [49]. The flavonols quercetin and kaempferol, as well as their glucosides, were also identified in *Helichrysum italicum* methanolic extracts (up to 0.015% and 0.0026%, respectively) [50]. The presence of flavones luteolin and apigenin in *Helichrysum italicum* ethanolic extracts, as well as the flavanone pinocembrin in methanolic extracts, was also reported; however, their extraction yields were not specified [3,125].

The flavonol gnaphaliin and flavanone pinocembrin, isolated from the methanolic extract of *Helichrysum italicum*, were able to inhibit the production of inflammatory leukotriene B4 in an in vitro model of calcium ionophore A23187-stimulated rat polymorphonuclear leukocytes by 94% and 96%, respectively, in comparison with the untreated control [21]. According to the authors, gnaphaliin, tiliroside, and pinocembrin (0.5 g) also reduced TPA-induced edema in the mice ears by 72, 80, and 81%, respectively (ID50 values of 210 μg/ear, 357 μg/ear, and 61 μg/ear, respectively). Tiliroside also diminished neutrophil infiltration by 88% [21]. An anti-inflammatory activity of naringenin (0.3 μM) in CD1 mouse, assessed as 43% inhibition of croton oil-induced ear edema relative to untreated control, was also observed [87]. Moreover, Shin et al. [126] observed inhibition of nuclear factor kappa B (NF-κB) activation by naringenin (10 uM) in colon HCT116 cells, which was assessed as inhibition of TNF-α-induced transcriptional activation.

Sala et al. [21] investigated the antioxidant properties of three flavonoids, gnaphalin, pinocembrin, and tiliroside, isolated from the aerial parts of *Helichrysum italicum*. Tiliroside exhibited the best DPPH scavenging potential (IC_50_ value of 6 μM), as well as significant inhibition of enzymatic and non-enzymatic lipid peroxidation (IC_50_ values of 12.6 and 28 μM, respectively). Tiliroside also exhibited superoxide-scavenging activity with an IC_50_ value > 100 μM. The superoxide-scavenging activity of naringenin was reported as well (IC_50_ value > 50 μM) [127].

In the study of Sun et al. [128], tiliroside significantly inhibited the main cytochrome P450 (CYP) enzymes present in the metabolism of clinically important drugs, in comparison with positive CYP inhibitors. Tiliroside was the most effective inhibitor of CYP2C9 (85%) with an IC_50_ of 10.2 ± 0.9 μM, followed by CYP2C8 (82.3%) with an IC_50_ value 12.1 ± 0.9 μM, and CYP3A4 (71.6%) with an IC_50_ value of 9.0 ± 1.7 μM. Takemura et al. [129] reported that naringenin also inhibited human CYP1A1, CYP1A2, and CYP1B1 enzymes (IC_50_ values of 15.17, 26.34, and 3.66 μM, respectively). Furthermore, Chen et al. [130] reported the antifungal activity of tiliroside (100 ug/disc) against *Ceratocystis paradoxa*, *Athelia rolfsii*, and *Alternaria mali* assessed as mycelial growth inhibition (GI) of 27.6, 22.4, and 55.6%, respectively. The same authors also reported cytotoxicity of tiliroside (20 mg/L) against cotton leafworm *Spodoptera litura* cells (GI 65%). In addition, the antiparasitic activity of tiliroside against *Entamoeba histolytica* (IC_50_ 17.45 μM) was observed [131]. Freitas et al. [132] reported the antileishmanial activity of tiliroside (841 uM) against *Leishmania amazonensis amastigote* (67.8%) and *Trypanosoma cruzi amastigote* (45%) as well. Tan et al. [133] also observed weak inhibition of HIV1 by tiliroside (IC_50_ < 200 μg/mL). On the other hand, Li et al. [134] reported that naringenin strongly inhibited His6-tagged HIV-1 integrase with an IC_50_ value of 1.7 μM. Moreover, the antifungal activity of naringenin against *Candida albicans* and *Cryptococcus neoformans* ATCC 90113 was reported at IC_50_ values of >50 μg/mL [135].

#### 4.1.3. Acetophenones and Tremetones

Acetophenones or methyl phenyl ketones are aromatic compounds that were first isolated in hydroxylated form from *Helichrysum italicum* methanolic extracts by Sala et al. [88]. Tremetones, also identified in *Helichrysum italicum* methanolic extracts in hydroxylated form, can be classified as benzofurans. Specifically, in the study of Sala et al. [88], six acetophenones and 12-hydroxytremetone (bitalin A) were isolated from the methanolic extract of *Helichrysum italicum* and then tested in two in vitro models and one in vivo model for their ability to inhibit arachidonic acid metabolism, and for evaluation of their antioxidative and anti-inflammatory potential. In the first in vitro model of calcium ionophore A23187-stimulated rat polymorphonuclear leukocytes, 4-hydroxy-3-(3-methyl-2-butenyl)acetophenone (100 μM) was able to reduce the production of leukotriene B4 by 95% (IC_50_ 24 μM) and 4-hydroxy-3-(2-hydroxy-3-isopentenyl)acetophenone (100 μM) reduced the production of leukotriene B4 by 44% (IC_50_ 111 μM). In the second in vitro model, only 4-hydroxy-3-(3-methyl-2-butenyl)acetophenone (100 μM) inhibited the activity of cyclooxygenase-1 (COX1) in calcium ionophore A23187-stimulated human platelets by 59%. Interestingly, none of the compounds exhibited scavenging activity against superoxide radicals. In the in vivo model, orally administered 4-hydroxy-3-(3-methyl-2-butenyl)acetophenone (150 mg/kg) reduced the carrageenan-induced edema in the mice paws by 51% after 1 h, by 71% after 3 h, and by 66% after 5 h. When the edema was induced by multiple injections of 2 μg TPA in mice ears, 4-hydroxy-3-(3-methyl-2-butenyl)acetophenone (0.5 mg) and 12-hydroxytremetone reduced the edema formation by 57%, and 71%, respectively [88]. The most effective compounds against PLA2-induced paw edema were 12-hydroxytremetone-12-O-β-D-glucopyranoside, 3-(2-hydroxyethyl)acetophenone-4-O-β-D-glucopyranoside and maltol β-D-O-glucopyranoside, which reduced the edema by 65, 57, and 52%, respectively [88].

Sala et al. [53] tested the anti-inflammatory activity of several acetophenones from dichloromethane, ethyl acetate, and butanol fractions of *Helichrysum italicum* methanolic extract. According to the results, 4-hydroxy-3-(2-hydroxy-3-isopentenyl)acetophenone isolated from the dichloromethane fraction proved to be the most active inhibitor of TPA-induced inflammation in mice ears with ID50 of 0.63 μmol/ear. Rigano et al. [58] first isolated a new acetophenone derivative gnaphaliol 9-O-propanoate together with known acetophenones, such as 1-[2-[1-[(acetyloxy) methyl]ethenyl]-2,3-dihydro-3-hydroxy-5-benzofuranyl]-ethanone and acetotrixymetone, from flowers of *Helichrysum italicum* subsp. *italicum*. A safe toxicological profile was confirmed for all three acetophenones, while only acetotrixymetone exhibited antioxidative activity. Interestingly, none of the compounds (1–30 μM) exhibited anti-inflammatory activity, since the LPS-induced increase in nitrite levels was not significantly modified.

#### 4.1.4. Pyrones

Arzanol, a prenylated phloroglucinyl α-pyrone heterodimer, was identified as the major anti-inflammatory compound in acetone extracts of aerial parts of *Helichrysum italicum* subsp. *microphyllum,* representing 0.32% of extraction yield [13]. According to Appendino et al. [10] arzanol represents a potent inhibitor of nuclear transcription factor NF-*κ*B activation with an IC_50_ value of 5 μM. Moreover, it was proven to inhibit the release of proinflammatory mediators in human peripheral monocytes such as IL-1*β* (IC_50_ 5.6 μM) and TNF-*α* (IC_50_ 9.2 μM), as well as IL-6, prostaglandin E2 (PGE2), and IL-8 with the IC_50_ values of 13.3, 18.7, and 21.8 μM, respectively. Bauer et al. [11] also investigated the effects of arzanol on the biosynthesis of prostaglandins and leukotrienes in vitro and in vivo. According to the authors, arzanol can inhibit the inducible microsomal prostaglandin E2 synthase (mPGE2), the formation of leukotriens in human neutrophilis, COX1 and 5-lipoxygenase (5-LOX) in vitro, with IC_50_ values ranging from 0.4 μM to 9 μM. It was also reported that the inhibition of PGE2 biosynthesis resulted from arzanol’s interference with mPGES rather than COX2. In vivo, arzanol (3.6 mg/kg) suppressed the carrageenan-induced inflammatory response in the pleural cavity of rats and significantly reduced exudate formation (59%), cell infiltration (48%), and levels of PGE2, leukotriene B4 (LTB4) and 6-keto prostaglandin F1 alpha (PGF1α) by 47, 31, and 27%, respectively. According to Rosa et al. [70], arzanol, isolated from *Helichrysum italicum* also possesses cytotoxic potential, as it selectively reduced viability of colon Caco-2 cells (55%) at a concentration of 100 µg/mL as well as in immortal HeLa (36%) and melanoma B16F10 (95%) cancer cell lines at the highest tested concentration of 200 µg/mL. Moreover, Appendino et al. [10] reported that arzanol inhibits the TNFα-induced HIV-1 replication in a T cell line in a concentration-dependent manner. Anti-HIV activity was further investigated by infecting Jurkat (T lymphocyte) cells with a pNL4-3 HIV-1 clone pseudotyped with the vesicular stomatitis virus (VSV) envelope, which can support HIV-1 replication. A pretreatment of Jurkat cells with increasing concentration of arzanol (5–25 μM) resulted in a concentration-dependent inhibition of viral replication (35–65%). Furthermore, in the study of Rosa et al. [71] the protective effect of arzanol in lipid peroxidation was investigated. Its antioxidant activity was tested against the Cu^2+^ ions-induced oxidative modification of lipid components in human low-density lipoprotein (LDL) and tert-butyl hydroperoxide (TBH)-induced oxidative damage in cell membranes. In vitro, LDL pretreatment with arzanol (50 μM) significantly protected lipoproteins from oxidative damage and exerted a remarkable reduction of polyunsaturated fatty acid and cholesterol levels (*p* < 0.001 versus oxidized control). At non-cytotoxic concentrations (25 μM and 50 μM), it also significantly protected kidney Vero cells and Caco-2 epithelial cells against TBH-induced oxidative stress. Rosa et al. [12] also confirmed that arzanol from *Helichrysum italicum* subsp. *microphyllum* did not exhibit toxicity in Vero cell cultures at any tested concentrations (0.5–40 μM). Tagliatela-Scafati et al. [13] evaluated the antibacterial activity of arzanol, coumarates, benzofurans, pyrones, and heterodimeric phloroglucinols isolated from *Helichrysum italicum* subsp. *microphyllum*. Only heterodimeric phloroglucinyl pyrone arzanol was efficient against multidrug-resistant *Staphylococcus aureus* strains, with MIC values of 1–4 μg/mL. In addition, Werner et al. [136] isolated and characterized two new arzanol derivatives from aerial parts of *Helichrysum italicum*, namely helitalone A, a dimer of substituted α- and γ-pyrone units, and helitalone B, a compound similar to arzanol with the isopropyl group replaced by an ethyl group. Antibacterial activities of isolated pyrone derivatives were tested against various Gram-positive and Gram-negative bacteria, but they did not exhibit any significant antibacterial effects at tested concentration of 20 μg/mL.

Arzanol can, therefore, act as a potential inhibitor of proinflammatory mediators, inflammatory enzymes, and HIV replication in T cells. Arzanol is also a potent natural antibacterial agent and antioxidant with a protective effect against lipid peroxidation in biological systems, and its diversity of action may well be utilized in cancer therapy.

#### 4.1.5. Triterpenes

Terpenes are a diverse class of aromatic organic compounds with a skeleton built from isoprene units, e.g., carbon atoms in the multiples of five (C5n). The most important terpenes from *Helichrysum italicum* extracts and essential oils can be divided into mono (C10), sesqui- (C15), and triterpenes (C30) based on the number of isopnene subunits. Ursolic acid is the only triterpene identified in acetone extracts of *Helichrysum italicum* in higher quantities (up to 0.40%) [13].

Liobikas et al. [137] reported the antioxidant activity of ursolic acid (1.6 ng/mL) in Wistar rat heart mitochondria, which was assessed as a reduction in H_2_O_2_ production by 55.6%. Anti-inflammatory activity of ursolic acid (10 mg/kg) against carrageenan-induced paw edema in Wistar albino rat model, after 4 h (75.17%) was also observed [138]. Ghosh et al. [139] reported antinociceptive activity (reduced sensitivity to pain) of ursolic acid (10 mg/kg) in Swiss albino *Mus musculus* model, which was assessed as 61.44% inhibition of formalin-induced paw licking, relative to untreated control, after 30 min. The antibacterial activity of ursolic acid against *Enterococcus faecalis* (MIC 16 μg/mL) was also reported [140]. Nguyen et al. [141] observed weak antiviral activity of ursolic acid (2.7 uM) against HIV1 3B-infected human leukemia CEM-SS cells, which was assessed as 22% inhibition of virus-induced cytopathic effect after 6 days. De Brum Vieira et al. [142] also reported the antiparasitic activity of ursolic acid against metronidazole-sensitive *Trichomonas vaginalis* (MIC 50 μM), while Freitas et al. [132] observed the antiparasitic activity of ursolic acid against *Trypanosoma cruzi* (IC_50_ 4 µM).

Kwon et al. [143] reported induction of apoptosis by ursolic acid (40 μM) in human prostate RC-58T/h/SA#4 cells, which was assessed as an increase in sub-G1 DNA content by 58.6% after 24 h. Ursolic acid (20.6 uM) also induced cell cycle arrest in human gastric AGS cells at sub-G0/G1 phase and G0/G1 phase by 86.53% and 33.2%, respectively, after 48 h [144]. Yang et al. [145] also observed weak antiproliferative activity of ursolic acid (100 μM) against rat liver HSC-T6 cells after 48 hrs (14.8%). Cytotoxicity of ursolic acid (50 μM) against human immortal HeLa cells and vaginal malignant melanoma HMVII cells were assessed as a reduction in cell viability by 50% and 60%, respectively, after 24 h [142]. In addition, ursolic acid (50 μM) demonstrated cytotoxicity against vaginal malignant melanoma HMVII cells by a 90% reduction in cell viability after 48 h. Wiemann et al. [146] reported cytotoxicity of ursolic acid against various human cancer cell lines, especially against colon HT-29 cancer cells (EC_50_ 10.6 μM) and human ovarian A2780 cancer cells (EC_50_ 11.7 μg/mL). Known biological effects of major bioactive compounds identified in extracts of *Helichrysum italicum* are summarised in Table 3.

## 5. Biological Effects of *Helichrysum italicum* Essential Oils

*Helicrysum italicum* essential oils are complex, yellow-colored, lipid-soluble liquids composed of volatile secondary plant metabolites characterized by a strong odor similar to curry [150]. Due to their antibacterial, antiviral, antifungal, and medicinal properties, as well as their pleasant fragrance, *Helichrysum italicum* essential oils are largely employed in agronomic, food [151], cosmetic [152], and perfume industries [153]. They are commercially used in perfumes, sanitary products, dentistry, agriculture, as food preservers and additives, and as antimicrobial, analgesic, sedative, anti-inflammatory, spasmolytic, as well as local anaesthetic remedies. *Helichrysum italicum* essential oils are very complex natural mixtures that can vary in composition and concentration of bioactive compounds according to climate, soil composition, plant organ, age, and vegetative cycle stage [150,154,155]. *Helichrysum italicum* essential oils are characterized by two or three major compounds at higher concentrations (20–70%), which determine the biological properties of the essential oils. Other compounds are present in trace amounts [150].

Voinchet and Giraud-Robert [9] reported that essential oils from *Helichrysum italicum* can significantly aid skin regeneration after cosmetic and reconstructive surgery and help with reduce inflammation, edema, bruising, and wound healing. Due to their antiallergenic properties, they can be helpful in the healing of asthma, hay fever, and eczema [156]. *Helichrysum italicum* essential oil is known to prevent skin aging and is, therefore, widely used in the formulations of antiaging creams and cosmetics. Recently reported in vitro anti-collagenase and anti-elastase activities of *Helichrysum italicum* essential oils support the use of the plant in the cosmetic industry [152]. *Helichrysum italicum* essential oil is also used in aromatherapy practice due to its ability to reduce couperose skin (red veins), hematoma, and thrombosis [23,156]. *Helichrysum italicum* essential oil, rich in neryl acetate, γ-curcumene, and α-pinene, showed significant anti-proliferative activity (*p* < 0.01 compared to vehicle control) in human dermal fibroblast culture (HDF3CGF). It also inhibited the production of collagen I and III, involved in tissue remodeling, which suggests great wound healing potential [157]. According to Conti et al. [15] the *Helichrysum italicum* essential oil (rich in neryl acetate, α-pinene, limonene and γ-curcumene) at the highest dosage of 300 ppm also induced high toxicity against asian tiger mosquito *Aedes albopictus* with a mortality rate of 100% (LC_50_ = 178.1 ppm). The promising insecticidal and repellent activity of *Helichrysum italicum* essential oil, rich in neryl acetate and neryl propanoate, against maize weevil *Sitophilus zeamais Motsch* (up to 85%) [37] as well as against housefly *Musca domestica* (LD50 value of 42 μg/adult) [158] has been reported.

Djihane et al. [14] isolated *Helichrysum italicum* essential oil from Algeria with predominate oxygenated sesquiterpenes α-cedrene (13.61%), α-curcumene (11.41%), and geranyl acetate (10.05%), and tested its antimicrobial and antifungal activities. The most sensitive bacterium was Gram-positive *Enterococcus cereus* ATCC 2035 with MIC and MBC values of 0.79 μg/mL. A minimum fungistatic concentration (MFC) and minimum fungicide concentration (MFC) of 6.325 μg/mL and 12.65 μg/mL, respectively, were obtained with the yeasts *Candida albicans* and *Saccharomyces cerevisiae*, whereas the four fungi were more resistant (MFC up to 50.6 μg/mL). In addition, Mastelic et al. [34] reported that the terpenoid fraction and its oxygen-containing compounds from Croatian *Helichrysum italicum* essential oil were the most effective against pathogenic yeast *Candida albicans* (MIC 5 μg/mL), as well as the Gram-positive bacteria *Staphylococcus aureus* (MIC 5 μg/mL). Recently, Staver et al. [78] reported that essential oil from Central Dalmatia (Croatia), rich in γ-curcumene, α-pinene and neryl acetate, possessed weak to moderate antimicrobial activity against *Staphylococcus aureus* and *Staphylococcus epidermidis* as the most sensitive bacterial strains, with MIC values of 1.6 mg/mL and 6.4 mg/mL, respectively. Ornano et al. [36] reported a strong cytotoxic effect of *Helichrysum italicum* subsp. *microphyllum* essential oil from Sardinia (rich in neryl acetate, 5-eudesmen-11-ol (rosifoliol), δ-cadinene, and γ-cadinene) on human malignant melanoma cells (A375) with an IC_50_ value of 16 μg/mL. When determining antioxidant activity using DPPH and ABTS assays, Weglarz et al. [39] observed that both methanolic extract and essential oil from the *Helichrysum italicum* herb indicated a higher potential than those obtained from the inflorescences (74.72, 61.38 63.81 and 58.59% for DPPH assay, respectively). However, when testing antimicrobial activity, the essential oil from inflorescences (rich in neryl acetate and nerol) possessed stronger bacteriostatic power than the herb essential oil (rich in neryl acetate and α-pinene). Gram-negative bacteria were less sensitive to both essential oils than Gram-positive strains, among which *Staphylococcus aureus* was the most susceptible (MIC 1 mg/mL, MBC 16 mg/mL). According to the authors, the differences between *Helichrysum italicum* raw materials should be considered before their specific industrial applications.

Giraud-Robert et al. [159] conducted a study on 60 patients who were chronic carriers of hepatitis B or C. Many essential oils, among which was *Helichrysum italicum* from Corsica (rich in neryl acetate, diones as well as curcumene), were used orally as a monotherapy or as a complement to allopathic treatment (bitherapy with interferon pegyl alpha-2a or alpha-2b (IFN-a) and ribavirin). When patients with hepatitis C were given bitherapy with essential oils, their condition improved by 100%. With essential oil monotherapy, there was an improvement in 64% of patients with hepatitis C and two patients with hepatitis B were cured. In addition, Nostro et al. [22] reported that genotoxicity of *Helichrysum italicum* essential oil in Vero cells appeared only at concentrations of 800 μg/mL. Idaomar et al. [160] proposed that the significant antigenotoxic effect of *Helichrysum italicum* essential oil against promutagen urethane might occur due to the interaction of its compounds with the cytochrome P450 enzymes, which are involved in the metabolic conversion of urethane, into ultimate carcinogenic metabolite vinyl carbamate epoxide. However, the molecular mechanisms remain unknown [161]. According to Foti et al. [162], healthy individuals did not display any adverse effects related to the utilization of the *Helichrysum italicum* essential oil. On the other hand, the authors reported the occurrence of allergic dermatitis in a 69-year-old non-atopic woman caused by hydrophilic and lipophilic fractions of the flowering tops.

Generally, the major compounds are found to reflect the biological features of the essential oils from which they are isolated [163]. However, it should be noted that *Helichrysum italicum* essential oils, similar to its extracts, represent complex mixtures of various bioactive compounds, which exert synergistic biological effects. The amplitude of biological effects is mostly dependent on the concentrations of bioactive compounds in essential oils and whether the compounds were tested alone or comprised in essential oils [150]. Cal et al. [164] suggest that the mixture of various compounds possesses an important role in defining the fragrance, density, texture, color, cell penetration, and cellular distribution of *Helichrysum italicum* essential oils.

### 5.1. Biological Effects of Major Bioactive Compounds from Helichrysum italicum Essential Oils

The main chemical compounds present in *Helichrysum italicum* essential oils can be divided into monoterpenes (C10) and sesquiterpenes (C15). The monoterpenes are formed from the coupling of two isoprene units (C10) and are the most representative terpenes, constituting 90% of the essential oils. The sesquiterpenes are formed from the assembly of three isoprene units (C15), and their structure and function are similar to those of the monoterpenes [150].

Various *Helichrysum italicum* essential oils from two main subspecies of *Helichrysum italicum*, namely *italicum* and *microphyllum,* have been intensively studied. Morone-Fortunano et al. [4] analyzed 20 *Helichrysum italicum* subsp. *italicum* genotypes from different locations in Italy and Corsica (France) and revealed that the essential oils contained mainly γ-curcumene (up to 41%), β-selinene (up to 38%), α-selinene (up to 26.5%), and neryl acetate (up to 32%). The concentrations of nerol and γ-eudesmol also reached appreciable amounts in some samples (up to 18.8% and 20.6%, respectively). Furthermore, Leonardi et al. [33] studied the composition of 21 *Helichrysum italicum* essential oil samples of subsp. *italicum* from seven locations of Elba Island (Tuscany, Italy). Monoterpene and sesquiterpene hydrocarbons accounted for 2.3–41.9% and 5.1–20.1% of the identified compounds, respectively. Essential oils from Elba Island (Italy) subsp. *italicum* were dominated by neryl acetate (up to 45.9%), followed by α-pinene (up to 32.9%), eudesm-5-en-11-ol (up to 17.2%), limonene (up to 12.9%) and nerol (up to 12.8%) [33]. Tuscan Archipelago Islands *Helichrysum italicum* essential oil subsp. *italicum* was also dominated by neryl acetate (up to 44.5%), followed by neryl propionate (up to 16.4%), γ-curcumene (up to 13.7%), and nerol (up to 7.6%) [44]. On the other hand, *Helichrysum italicum* subsp. *italicum* essential oil sample from Cilento (Italy) was dominated by iso-italicene epoxide (16.8%) [165]. According to Bianchini et al. [166] subsp. *italicum* essential oil samples from Tuscany contained mainly α-pinene (up to 53.5%) and neryl acetate (up to 22%), followed by β-selinene (up to 12.5%) and β-caryopyllene (up to 11%), while the sample from Corsica was dominated by neryl acetate (up to 38.9%) followed by neryl propionate (up to 5.9%) [166]. In another study of Bianchini et al. [167], the characterization of Corsican essential oils subsp. *italicum* also identified neryl acetate as a predominant compound, with amounts from 15.8% (from plants in the stage of early shoots) to 42.5% (in full flowering period). Interestingly, *Helichrysum italicum* essential oil subsp. *italicum* from Greek island of Amorgos was characterized by a high content of geraniol (35.59%) and a significant amount of geranyl acetate (20.76%) and nerolidol (11.86%) [168].

According to Morone-Fortunato et al. [4], three different chemotypes were observed in subsp. *italicum*:

(a) genotypes rich in nerol and its esters;

(b) genotypes with a dominance of β and α-selinene;

(c) genotypes with high amounts of γ-curcumene.

Furthermore, essential oils subsp. *microphyllum* (Willd.) Nyman from Sardinia were mostly dominated by neryl acetate (26–35.6%) and nerol (9.1–14.4%) [63,169,170], while neryl propionate (up to 11.4%), γ-curcumene (up to 18.2%), and eudesm-5-en-11-ol (up to 23.5%) were also present in significant amounts. Melito et al. [27] examined 146 *Helichrysum Italicum* subsp. *microphyllum* genotypes from the seaside (0–60 m above the sea level) and mountains (600–1250 m above the sea level) in Sardinia to prove the influence of altitude and climate on the *Helichrysum italicum* essential oil composition. The results showed that there is a correlation between the habitat type and the secondary metabolite production based on significantly (*p* < 0.0001) different essential oil compositions between both habitats. Considering the importance of climatic factors on the chemical composition of the essential oil, the quantity of nerolidol was correlated with the mean winter temperature, while italicene, bergamotene, nerol, and curcumene were positively correlated with spring and summer percipitation. Similarly, two studied genotypes of *Helichrysum italicum* subsp. *microphyllum* from Corsica were rich in neryl acetate (up to 55.7%), and also contained appreciable amounts of neryl propionate (up to 12.7%) [6]. On the other hand, *Helichrysum italicum* subsp. *microphyllum* essential oil from Crete contained mainly sesquiterpenes β-selinene (up to 17.2%) and γ-curcumene (up to 13.7%) followed by α-selinene (up to 5.39%) [45].

It must be noted that many authors did not specify the subspecies of *Helichrysum italicum* from which the studied essential oils were obtained. For example, Croatian oil samples (subsp. not specified) were dominated by neryl acetate as a major compound (11.5%) [34], while a surprisingly lower content of neryl acetate (up to 9.02%) was present in *Helichrysum italicum* essential oils from the Croatian Adriatic coast (subsp. not specified), where α-pinene (up to 29.9%), and α-curcumene (up to 28.64%) were determined as major compounds [171]. In a recent study, Oliva et al. [77] analyzed the composition of *Helichrysum Italicum* essential oil (subsp. not specified) from Montenegro. According to the results, essential oil from the liquid phase possessed high amounts of sesquiterpenes β-eudesmene (21.65%), and β-bisabolene (19.90%), as well as monoterpenes α-pinene (16.90%) and neryl acetate (10.66%). On the other hand, the vapor phase was enriched with monoterpene hydrocarbons fraction with α-pinene (78.76%) as the major compound.

**Figure 4:**
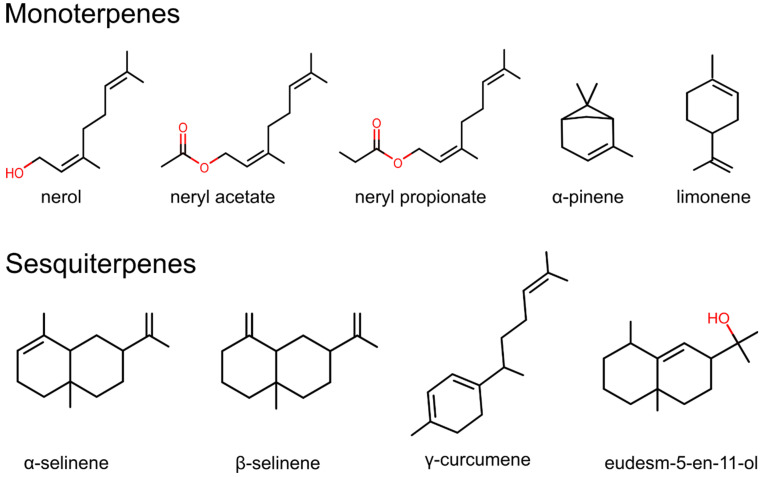
Major bioactive compounds from *Helichrysum italicum* essential oils.

It can be concluded that *Helichrysum italicum* essential oils exhibit various compositions depending on the geographical location where the plant grows, the sub-species, acidity, and type of soil, as well as the developmental stage of the plant. Due to different chemical compositions, essential oils from various sub-species and geographical locations may possess distinct biological effects. Hladnik et al. [172] revealed the complete chloroplast genome of *Helichrysum italicum* subsp. *italicum* sampled in the North Adriatic Region. The chloroplast genome contained 131 genes (85 protein-coding genes, 36 transfer RNA genes, 8 ribosomal RNA genes, and 2 partial genes) and its length was 152,431 bp. According to the authors, these findings could be used for the development of reliable molecular markers for future genetic studies of *Helichrysum italicum*. There are numerous research articles on *Helichrysum italicum* biochemical diversity, however, only a few are related to its genetic diversity and the relationship between genotypes and chemotypes [31]. In the following subsections, we summarise the already recognized biological effects of the major bioactive compounds identified in the *Helichrysum italicum* essential oils, which are presented in Figure 4.

#### 5.1.1. Monoterpenes

Based on the number of isoprene subunits, the most important terpenes from *Helichrysum italicum* essential oils belong to monoterpenes (C10) and sesquiterpenes (C15). Monoterpenes and sesquiterpenes from *Helichrysum italicum* essential oils also contain different functional groups and can be predominantly classified as alcohols (e.g., nerol, eudesm-5-en-11-ol) and esters (e.g., neryl acetate, neryl propionate).

Nerol and its derivatives are largely employed as cosmetic ingredients due to their sweet rose fragrance. The richest natural sources of monoterpene nerol include rose, palmarosa, and citronella as well as *Helichrysum italicum* essential oils. Its esters (nerol acetate in particular as well as nerol propionate) are also commonly encountered as major compounds in *Helichrysum italicum* essential oils from Italy and France (up to 18.8%, 55.7%, and 16.4%, respectively) [4,6,33,44,169]. In the study of Cordali et al. [173], nerol (10 μL) showed insecticidal activity against the first, second, and third-instar larval stage of *Leptinotarsa decemlineata*-infested potato leaves assessed as mortality relative to untreated control after 96 h (56.7%, 56.7%, and 80%, respectively). Ramos Alvarenga et al. [174] reported that nerol also possesses antimicrobial activity against *Mycobacterium tuberculosis* H37Rv at a MIC value of 128 μg/mL. Moreover, nerol was reported to possess acaricidal activity against *Psoroptes* cuniculi, which was observed at inhalation of 3 μL (83.3%) and 6 μL (100%) of nerol after 24 h [175]. The same authors also conducted a direct contact assay where nerol showed 100% acaricidal activity against *Psoroptes cuniculi* at 0.125, 0.25, and 1% dilution in physiological saline after 48 h. The repellent activity of nerol (0.2 uL/cm^2^) against *Tribolium castaneum* (red flour beetle) was also assessed as induction of repellency measured 2 h and 4 h after exposure (98% and 95%, respectively) [176]. In the study of Kordali et al. [173] neryl acetate (20 μL) showed lower insecticidal activity than nerol against the first, second, and third-instar larval stage of *Leptinotarsa decemlineata*-infested potato leaves, which was assessed as mortality relative to untreated control after 96 h (10, 6.7, and 46.7%, respectively). According to Ortar et al., [177] neryl acetate also has agonist activity against rat transient receptor potential cation channel, subfamily A, member 1 (TRPA1) expressed in human embryonic kidney HEK293 cells, which was assessed as inhibition of the increase in intracellular Ca^2+^ concentration (IC_50_ 21.2 μM).

α-pinene is the most abundant terpene in nature, which occurs in the essential oils of *Pinus palustris* Mill. at concentrations of up to 65%, *Pinus caribaea* at concentrations up to 70% [178] and *Helichrysum italicum* at concentrations up to 53.5% [33,34,166,171]. Nowadays, α-pinene is used in the production of gin [179]. Burits et al. [180] reported the potent antioxidative activity of pure α-pinene in the DPPH assay (IC_50_ value of 0.78 μL/mL) as well as emphasized its potential to inhibit lipid peroxidation (IC_50_ value of 0.51 μL/mL). De-Oliveira et al. [181] demonstrated that (–)-α-pinene and (+)-α-pinene modulate hepatic mono-oxygenase activity CYP2B1, which catalyzes biotransformation of promutagens or procarcinogens into genotoxic chemical carcinogens (IC_50_ value of 0.087 μM and 0.089 μM, respectively). Lorente et al. [182] demonstrated the anti-inflammatory activity of α-pinene (80 mg/kg) against carrageenan-induced plantar edema in Wistar rat paw (26.2% edema reduction). Rufino et al. [183] showed the anti-inflammatory activity of α-pinene (200 μg/mL) against human primary chondrocytes, which was determined as 40.6% inhibition of IL-1β-induced NO production relative to a IL-1β-treated control. α-pinene showed weak antimicrobial activity against other tested strains, namely *Candida albicans*, *Staphylococcus epidermidis*, *Pseudomonas aeruginosa*, *Escherichia coli* and *Staphylococcus aureus* (MIC > 900 μL/mL) [184].

Limonene, the main constituent of the citrus essential oil of sweet orange peel oil (*Citrus sinensis, Rutaceae*), is frequently present in considerable amounts in the *Helichrysum italicum* essential oil as well (up to 12.9%) [33,166,167]. Monocyclic monoterpene (+)- and (−)-limonene enantiomers are extensively used as fragrances in household cleaning products, in the cosmetic industry in creams, perfumes, and soaps, in the food industry as flavor additives for food, and as industrial solvents. According to Schnuch et al. [185], limonene belongs to the third group (Group III) of substances that are considered extremely rare sensitizers, and may even be considered as non-sensitizers (upper confidence interval (CI) of less than 0.5%). However, it must be noted that limonene can become an allergen after substantial air oxidation [186]. In the study of Souza et al. [187] the anti-inflammatory activity of limonene in the LPS-induced pleurisy mouse model was investigated. After oral administration of pure limonene, a significant reduction of LPS-induced cell migration was observed. Pure limonene also reduced the production of NO by 50% and inhibited γ-interferon by 86% at a dose of 25 μg/well. De-Oliveira et al. [181] demonstrated that d-limonene modulates hepatic monooxygenase activity of CYP2B1 enzyme (IC_50_ value of 0.19 μM), which catalyzes the biotransformation of procarcinogens. Wilkins et al. [188] identified d-limonene as effective in the treatment of gastroesophageal reflux disorder. A double-blind, placebo-controlled trial was conducted with 13 patients. After 14 days 86% of patients who took d-limonene were asymptomatic. In the placebo group, only 29% of patients reported relief of symptoms after 14 days.

#### 5.1.2. Sesquiterpenes

α and β-selinene are ubiquitous sesquiterpene hydrocarbons present as the major compounds in *Helichrysum italicum* subsp. *italicum* essential oil from Italy and Corsica (up to 26.5% and 16.7%, respectively) [4,45]. They possess sweet woody and herbaceous fragrances, which play an important role in chemical ecology as pheromones [179]. Moreover, γ-curcumene and eudesm-5-en-11-ol are sesquiterpenes, which have been identified as major compounds in the essential oils of *Helichrysum italicum* subsp. *italicum* from Italy and Corsica (up to 41% and 17.2%, respectively) [45]. The biological activities of individual major sesquiterpenes from *Helichrysum italicum* essential oils currently remain unexplored. Sesquiterpenes, therefore, represent interesting candidates for further research. Known biological effects of major bioactive compounds from essential oils of *Helichrysum italicum* are summarised in Table 4.

## 6. Encapsulation of *Helichrysum italicum* Extracts, Essential Oils and Individual Bioactive Compounds

Low absorption and bioavailability represent the main obstacles to the successful delivery of natural polyphenols from *Helichrysum italicum* extracts and essential oils from the gastrointestinal tract to the targeted tissues in vivo. To improve bioavailability, absorption, solubility, and rapid metabolic degradability of polyphenols, various drug delivery systems, such as nanoparticles, emulsions, and liposomes have been intensively studied [192,193,194]. Encapsulation (microencapsulation, nanoencapsulation) is a simple and cost-effective method in which bioactive compounds are coated or entrapped into cell wall material. Polysaccharides, derived from animals (chitosan), algae (alginate, carrageenan), plants (pectin, starch, cellulose, hyaluronate), and bacteria (dextran and xanthan gum) are commonly used for bioactive compound encapsulation. *Helichrysum italicum* extract was successfully encapsulated into various alginate-protein matrices, which served as carriers for the formulation of biodegradable edible films of immortelle [195]. Chitosan is also considered as an effective delivery system for polyphenolic compounds [196,197] and is often combined with natural polysaccharides, such as alginate, to form complexes [198,199,200].

Nowadays, liposomes are receiving increasing attention as one of the most promising carriers of various bioactive polyphenolic compounds, as they exhibit exceptional biocompatibility, biodegradability, non-toxicity, non-immunogenicity, improved targeted delivery, and successfully protect polyphenolic compounds from light and degradation processes [201]. Liposomes, vesicles that consist of one or more phospholipid bilayers, possess significant potential in the cosmetic and food industries due to minimal adverse effects [201]. Successful encapsulation of biologically active polyphenolic compounds [202], extracts [203,204], and essential oils [205] obtained from different natural materials into liposomes was recently reported in several studies. Pharmaceutical and cosmetic formulations with liposomes incorporating bioactive compounds allow better bioavailability of bioactive compounds, thereby increasing their efficacy [206]. Liposomes with encapsulated extracts of various herbs and spices exhibited excellent inhibitory effects against various tested bacterial strains, which was even higher than in the case of tested pure extracts [207]. Liposomes can also protect natural polyphenols from *Helichrysum italicum* against metabolic degradation, enhance their beneficial effects in the target tissues, and amplify their antioxidative, anti-inflammatory, antibacterial, and anticarcinogenic effects, which is vitally important in the treatment of various diseases. In addition, nanoparticle drug delivery systems using liposomes as well as natural polysaccharides (such as chitosan, alginate, pectin, cellulose, and xanthan gum) represent promising alternatives to magnetic metal-based nanoparticles due to their reduced toxicity, higher biocompatibility, and improved targeted delivery. Future studies should, therefore, focus on the incorporation of bioactive compounds from *Helichrysum italicum* into liposomes and polysaccharides. This will represent an important novelty for cosmetic formulations and dietary supplements.

## 7. Conclusions and Future Perspectives

In this review, we identify major bioactive compounds of *Helichrysum italicum* extracts and essential oils, which exhibit promising antioxidant, anti-inflammatory, antimicrobial, antiviral, insecticidal and anticarcinogenic properties without harmful side effects on humans and animals. Moreover, modern extraction and distillation techniques, as well as analytical methods for efficient isolation and characterization of *Helichrysum italicum* extracts and essential oils, together with methods for determining their antioxidative, antimicrobial, anti-inflammatory, and anticarcinogenic activities, are presented.

It can be concluded that prenylated phloroglucinyl α-pyrone arzanol, flavonoids gnaphaliin, pinocembrin, and tiliroside, as well as acetophenone 4-hydroxy-3-(3-methyl-2-butenyl)acetophenone represent the major bioactive compounds in non-volatile extracts, whereas the volatile essential oils showed a dominance of monoterpenes, nerol and its esters, α-pinene, and limonene, as well as the sesquiterpenes α-selinene, β-selinene, and γ-curcumene. Extraction with organic solvents, such as ethanol, methanol and acetone, is most frequently employed to obtain non-volatile *Helichrysum italicum* extracts, whereas hydrodistillation and steam distillation are preferred for the isolation of volatile essential oils. However, there is an evident lack of studies addressing the biological activities of supercritical extracts of various *Helichrysum italicum* subspecies. Therefore, the isolation of polyphenolic compounds by supercritical CO_2_ with the addition of cosolvents, as well as their identification with HPLC, should be further explored.

Future studies should focus on liposomes and polysaccharide nanoparticles as drug delivery systems for *Helichrysum italicum* extracts and essential oils because they provide reduced toxicity, higher biocompatibility, improved bioavailability, and targeted delivery of bioactive polyphenolic compounds. Moreover, the identification of subspecies, the key genes controlling the biosynthesis of bioactive secondary metabolites as well as reliable discrimination between ornamental, cultivated, and wild plants is crucial for further applications of *Helichrysum italicum* in food, cosmetic and pharmaceutical industries. An under-explored area is also the cultivation management of the *Helichrysum italicum* species, including sowing requirements, plant density, irrigation, fertilization, harvesting, and its impact on the extraction and essential oil yields, quality, and composition.

**Figure 5:**
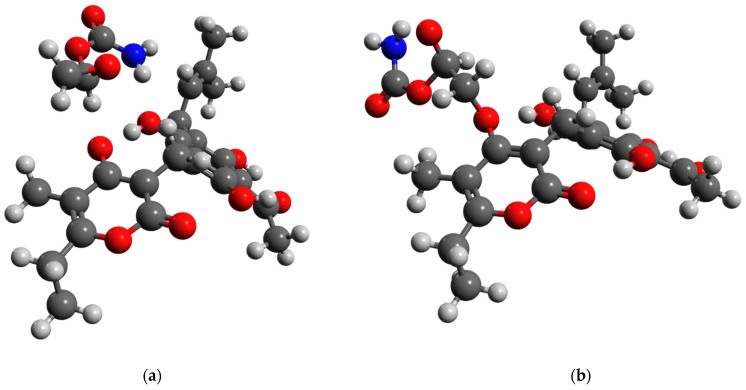
Quantum-mechanical models of (**a**) reactants and (**b**) transition state structure of the most studied compound from the *Helichrysum italicum* extracts–arzanol with vinyl carbamate epoxide obtained with Hartree-Fock method and flexible 6-311++G(d,p) basis set. Carbon atoms are depicted in grey, oxygen in red, nitrogen in blue and hydrogen atoms in white.

Several in vitro studies have shown that polyphenols, namely arzanol, gnaphaliin, and tiliroside from *Helichrysum italicum* have the potential for cancer prevention. Due to the complicated and intertwined mechanisms involved in cancer initiation and progression, in silico studies are required to reveal specific molecular mechanisms of *Helichrysum italicum* polyphenols. In silico quantum-mechanical simulations performed by our research group [208,209] represent a safe approach to reveal cancer-preventive mechanisms of bioactive polyphenols, such as arzanol from *Helichrysum italicum*, against various ultimate chemical carcinogens, including the metabolic product of urethane, vinyl carbamate epoxide, found in fermented foods (Figure 5). Mechanistic insights into *Helichrysum italicum* polyphenol interactions with human, bacterial, fungal, and viral proteins, which are crucial for the design and optimization of novel drugs, can be revealed by our in house developed inverse molecular docking protocol [210,211] as well as by extensive molecular dynamics simulations coupled with free-energy calculations [212,213].

It can be concluded that *Helichrysum italicum* possesses various beneficial health effects, and has the potential for applications in the cosmetic, pharmaceutical, and food industries, as well as in the development of novel antimicrobial, antiviral, and insecticidal agents. This review provides a complete overview of the already recognized biological effects of major bioactive compounds present in *Helichrysum italicum* extracts and essential oils and can, therefore, guide future research on this important everlasting plant.

## Figures and Tables

**Table 2:** Overview of *Helichrysum italicum* major bioactive compounds obtained by supercritical CO_2_ extraction.

Major Compounds	Extraction Temperature (°C)	Extraction Pressure (bar)	Extraction Time (h)	Yield (%)	Identification Method	References
**Monoterpenes** α-Pinene, nerol, neryl acetate, and neryl propanoate **Sesquiterpenes** α–Selinene, β-selinene, γ-curcumene, nerolidol, acetate *, widdrol *, β-eudesmol* eudesm-5-en-11-ol *, waxes *	40–60	100–200	1.5	1.37–4.1	GC-FID, GC-MS	[60]
	40	150	1.7	5.7	GC-FID, GC-MS	[62]
	50	90	2–4	0.4–1	GC-MS	[63]
	40	80–350	3	0.35	GC, GC-MS	[64]
	40	90–120	-	0.36–0.60	GC-FID, GC-MS	[65]
	40	350	5.5	3.60 ± 0.23 7.14 ± 0.58 *	GC-FID, GC-MS	[66]
**Polyphenolic compounds** Pyrogallol, chlorogenic acid derivatives, naringenin, pinocembrin, arzanol *, gentisic acid *, caffeic acid *, luteolin *, tiliroside *, quercetin *, kaempferol *, and apigenin *	40	350	5.5	3.60 ± 0.23 7.14 ± 0.58 *	HPLC-MS	[66]
Scopoletin	35.86–64.14	79.3–220.7	1.5	0.43–6.31	HPLC, UV-VIS	[67]

* SFE with the addition of cosolvent ethanol.

**Table 3:** Known biological effects of bioactive compounds from *Helichrysum italicum* extracts.

Compound	*Helichrysum italicum* Subspecies	Extraction Yield from Starting Plant Material	Known Biological Effects
**Phenolic acids**			
Caffeic acid	*Picardii* Subspecies not specified	up to 0.77% [113] up to 0.0067% [49]	Antioxidant activity [114], anti-inflammatory activity [86,117], histone deacetylase inhibition [116], anticancer activity [118], neuroprotective activity [119], antiviral (anti-HIV) activity [123], antibacterial activity [120,121], antifungal activity [122]
Chlorogenic acid	*Picardii* Subspecies not specified	up to 0.015% [113] up to 0.104% [49]	Antioxidant activity [104], anti-inflammatory activity (inhibition of COX2) [107], anticarcinogenic properties (inhibition of cell proliferation) [108], antibacterial activity [109,110], antifungal activity [111]
**Flavonoids**			
Gnaphaliin	Subspecies not specified	up to 0.03% [54]	Antioxidant activity [21], anti-inflammatory activity [21]
Tiliroside	Subspecies not specified	up to 0.0063% [54]	Antioxidant activity [21,115], anti-inflammatory activity [21], inhibition of CYP enzymes [128], antifungal activity [130], antiparasitic activity [131,132], antiviral (anti-HIV) activity [133]
Naringenin	Subspecies not specified	up to 0.023% [49]	Antioxidant activity [115], anti-inflammatory activity [87,126], inhibition of CYP enzymes [129], antibacterial activity, antifungal activity [135], antiviral (anti-HIV) activity [134]
Pinocembrin	Subspecies not specified	Not specified [125]	Antioxidant activity [21], anti-inflammatory activity [21] antibacterial activity [147], neuroprotective activity [148]
**Acetophenones**			
4-Hydroxy-3-(3-methyl-2-butenyl) acetophenone	Subspecies not specified	3.64% [53]	Anti-intiiflammaroty activity [88], inhibition of cyclooxygenase-1 (COX1) [88]
4-Hydroxy-3-(2-hydroxy-3-isopentenyl)acetophenone	Subspecies not specified	0.04% [53]	Anti-inflammatory activity [53]
**Tremetones**			
12-Hydroxytremetone	Subspecies not specified	0.18% [53]	Anti-inflammatory activity [88]
**Pyrones**			
Arzanol	*Microphyllum*	up to 0.32% [13]	Antioxidant activity [71], anti-inflammatory activity (potential inhibitor of pro-inflammatory mediators [10] and inflammatory enzymes COX1, COX2, and 5-LOX) [11], cytotoxic activity against cancer cells [70], antibacterial activity [13], antiviral (anti-HIV) activity [10]
**Triterpenes**			
Ursolic acid	*Microphyllum*	up to 0.40% [13]	Antioxidant activity [137], anti-inflammatory activity [138], anticancer activity, induction of apoptosis [143], cell cycle arrest [144], antiproliferative activity [145], cytotoxicity to cancer cells [142,146,149], antibacterial activity [140], antiparasitic activity [132,142], antiviral (anti-HIV) activity [141]

**Table 4:** Known biological effects of major bioactive compounds from *Helichrysum italicum* essential oils.

Compound	*Helichrysum italicum* Subspecies	Compounds Content in Essential Oil	Know Biological Effects
**Monoterpenes**			
Nerol	*Microphyllum* *Italicum*	up to 14.4% [169] up to 18.8% [4]	Insecticidal activity [173], antimicrobial activity [174], acaricidal activity [175], repellent activity [176], food additive [189]
Neryl acetate	*Microphyllum* *Italicum*	up to 55.7% [6] up to 45.9% [33]	Insecticidal activity [173], repellent activity [176], the agonist of TRPA1 [177], food additive [190]
Neryl propionate	*Microphyllum* *Italicum*	up to 11.4% [170] up to 16.4% [44]	Food additive [190]
α-Pinene	*Italicum*	up to 53.5% [166]	Antioxidative activity [180], anti-inflammatory activity [182,183], inhibition of CYP enzymes [181], antimicrobial activity [184], food additive [191]
Limonene	*Italicum* *Microphyllum*	12.9% [33] up to 7% [169]	Anti-inflammatory activity [187], gastroprotective effects [188], inhibition of CYP enzymes [181], food additive [191]
**Sesquiterpenes**			
α-Selinene	*Microphyllum Italicum*	up to 5.4% [45] up to 26.5% [4]	Pheromone [179]
β-Selinene	*Microphyllum Italicum*	up to 17.2% [45] up to 38% [4]	Pheromone [179]
γ-Curcumene	*Microphyllum Italicum*	up to 18.2% [170] up to 41% [4]	Unknown
Eudesm-5-en-11-ol	*Italicum Microphyllum*	up to 17.2% [33] up to 23.5% [169]	Unknown

## Data Availability

All data are included in the article.

## Abbreviations

5-LOX	5-lipoxygenase
ABTS	2,2’-azino-bis(3-ethylbenzothiazoline-6-sulphonic acid)
AChE	Acetylcholinesterase
AD	Alzheimer’s disease
CI	Confidence interval
COSY	Correlated spectroscopy
COX1	Cyclooxygenase 1
COX2	Cyclooxygenase 2
CYP	cytochrome P450 enzymes
DAD	Diode array detection
DNA	Deoxyribonucleic acid
DPPH	2,2-diphenyl-1-picrylhydrazyl
EC50	Half maximal effective concentration
EI-MS	Electron ionization mass spectrometry
EO	Essential oil
FID	Flame ionization detector
GC	Gas chromatography
GC-MS	Gas chromatography-mass spectrometry
GI	Growth inhibition
HDAC	Histone deacetylases
HIV	Human immunodeficiency virus
HPLC	High performance liquid chromatography
HRESIMS	High-resolution electrospray ionisation mass spectrometry
HSV	Herpes simplex virus
IC50	The half maximal inhibitory concentration
ID50	Infectious dose 50
IL-1β	Interleukin-1beta
IL-6	Interleukin-6
IL-8	Interleukin-8
IL-12	Interleukin-12 subunit p40
LC50	Median lethal concentration
LD50	Median lethal dose
LDL	human low density lipoprotein
LPS	Lipopolysaccharide
LTB4	leukotriene B4
ITS1/2	Internal transcribed spacer 1 and 2
MBC	Minimum bactericidal concentration
MECC	Micellar electrokinetic capillary chromatography
MIC	Minimum inhibitory concentration
mRNA	Messenger ribonucleic acid
mPGES	Microsomal PEG2 synthase
MTS	3-(4,5-dimethylthiazol-2-yl)-5-(3-carboxymethoxyphenyl)-2-(4-sulfophenyl)-2H-tetrazolium
MTT	3-(4,5-dimethylthiazol-2-yl)-2,5-diphenyltetrazolium bromide
NF-κB	Nuclear factor kappa B
NMR	Nuclear magnetic resonance
NO	Nitric oxide
PTP1B	Protein tyrosine phosphatase 1B
PGE2	Prostaglandin E2
PGF1α	Prostaglandin F1 alpha
PLA2	Phospholipase A2
SFE	Supercritical fluid extraction
sub-MIC	Sub-minimum inhibitory concentration
TBH	Tert-butyl hydroperoxide
TLC	Thin-layer chromatography
TNF-α	Tumor necrosis factor alpha
TPA	12-O-Tetradecanoylphorbol-13-acetate
TRPA1	Transient receptor potential cation channel, subfamily A, member 1
UV-VIS	Ultraviolet–visible spectroscopy
VSV	Vesicular stomatitis virus
